# Zika Virus in Rectal Swab Samples

**DOI:** 10.3201/eid2505.180904

**Published:** 2019-05

**Authors:** Camila Helena Aguiar Bôtto-Menezes, Armando Menezes Neto, Guilherme Amaral Calvet, Edna Oliveira Kara, Marcus Vinícius Guimarães Lacerda, Marcia da Costa Castilho, Ute Ströher, Carlos Alexandre Antunes de Brito, Kayvon Modjarrad, Nathalie Broutet, Patrícia Brasil, Ana Maria Bispo de Filippis, Rafael Freitas Oliveira Franca, ZIKABRA Study Team

**Affiliations:** Amazonas State University, Manaus, Brazil (C.H.A. Bôtto-Menezes);; Tropical Medicine Foundation Doctor Heitor Vieira Dourado, Manaus (C.H.A. Bôtto-Menezes, M.V.G. Lacerda, M. da Costa Castilho);; Institute Aggeu Magalhães, Recife, Brazil (A.M. Neto, C.A. Antunes de Brito, R.F.O. Franca);; Evandro Chagas National Institute of Infectious Diseases, Rio de Janeiro, Brazil (G.A. Calvet, P. Brasil);; World Health Organization, Geneva, Switzerland (E.O. Kara, U. Ströher, N. Broutet);; Oswaldo Cruz Foundation, Manaus (M.V.G. Lacerda);; Walter Reed Army Institute of Research, Silver Spring, Maryland, USA (K. Modjarrad);; Oswaldo Cruz Institute, Rio de Janeiro (A.M. Bispo de Filippis)

**Keywords:** Zika virus, sexual transmission, virus persistence, infection, viruses, rectal swab, Brazil

## Abstract

We detected Zika virus RNA in rectal swab samples from 10 patients by using real-time reverse transcription PCR, and we isolated the virus from 1 patient. The longest interval from symptom onset to detection was 14 days. These findings are applicable to diagnosis and infection prevention recommendations.

In early 2015, Zika virus was identified in Brazil and spread across nearly the whole continent, affecting thousands of persons (*1*). This outbreak was associated with microcephaly and other congenital abnormalities resulting from infection of the mother during pregnancy (*2*). For different periods after infection, Zika virus RNA can be found in diverse body fluids such as saliva, amniotic fluid, urine, cerebrospinal fluid, blood, semen, and tears (*3*); the longest period of viral RNA shedding has been identified in semen (>1 year after symptom onset) (*4*). 

Although Zika virus RNA has been detected in different body fluids, we found only 1 report of Zika virus elimination through feces from 1 naturally infected person (*5*). Experimentally, Zika virus is able to infect mice and adult macaques (*6*) through the anorectal mucosa, leading to detectable viremia with subsequent testicular damage and congenital defects in the offspring of pregnant mice (*5*). These findings indicate that the anorectal mucosa may represent an infection route for Zika virus. However, whether Zika virus can be detected in the anorectal mucosa of naturally infected human patients remains largely unknown. To clarify the kinetics of Zika virus infection across biological compartments and to devise rational measures for preventing transmission of the virus, in July 2017 we began a cohort study of men and women >18 years of age with Zika virus infection in Brazil; the study will continue until mid-2020. Written informed consent was obtained from all enrolled participants.

## The Study

To assess the persistence of Zika virus in different body fluids of persons with confirmed infection, we conducted a multicenter prospective cohort study (the ZIKABRA Study). Laboratory confirmation of infection was based on real-time reverse transcription PCR (rRT-PCR) performed on samples (urine, blood, or both) from persons in whom a rash developed <48 hours after initial symptom onset. We also invited household contacts or sex partners to participate in the study and enrolled those with positive rRT-PCR results for Zika virus. Full details about the study protocol, including ethics approval, are described by Calvet et al. (*7*). 

We collected samples (saliva, blood, urine, vaginal, and rectal swabs) at specific intervals from patients identified as Zika virus positive after the screening visit (Figure). Vaginal and rectal swab samples were diluted in 1 mL of sterile Hank’s Balanced Salt Solution (ThermoFisher Scientific, https://www.thermofisher.com) and stored at −80°C until processing. After collection, specimens were kept refrigerated and transported within 2 hours to the laboratory, where they were maintained at −80°C. Zika virus detection was performed by rRT-PCR by processing 200 μL of each specimen for RNA extraction through an automated nucleic acid purification platform by using the Maxwell 16 Viral Total Nucleic Acid Purification Kit (Promega Corporation, https://www.promega.com) in a final volume of 70 μL. For rRT-PCR, we used the commercially available ZDC Kit from Instituto de Tecnologia em Imunobiológicos Biomanguinhos, approved by Agência Nacional de Vigilância Sanitária/ANVISA (registry no. 80142170032; https://www.bio.fiocruz.br). We considered positive those samples that displayed positive amplification in the ZDC Kit internal control reaction (which consists of an RNA virus–like particle individually added to each specimen before RNA extraction) and those samples in which the target amplification was detected within 38 amplification cycles, as previously described (*8*). 

**Figure:**
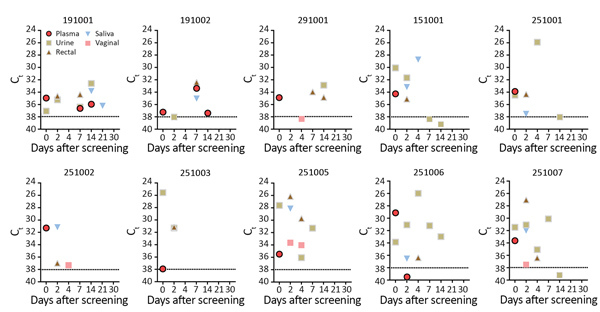
Detection of Zika virus RNA in human biological specimens from 8 patients, according to C_t_ and days after disease onset. Patient identification numbers above charts correspond to numbers in the Table. Horizontal dashed lines indicate real-time reverse transcription PCR cutoff C_t_ of 38. Disease onset is day 0 (screening visit), defined after interviewing patients about symptoms. C_t_, cycle threshold.

We report 10 Zika virus–infected patients from 2 locations in Brazil. Of these, 3 patients were identified in Recife, northeastern Brazil, and 7 were from Manaus, northern Brazil. The targeted Zika virus amplicons were found in the plasma of 9 patients and in the urine of 7 patients, all tested at screening visits. We also tested all plasma and urine samples for dengue and chikungunya virus by rRT-PCR; results were negative. Of the 10 Zika virus–infected patients, 9 were symptomatic and 1 (a Zika virus–positive household contact) was asymptomatic at enrollment but subsequently reported muscular weakness and irritability on day 5 and arthralgia on day 7 after Zika virus identification. Median patient age was 31.5 years; 7 patients were nonpregnant women, and 3 were men (Table). All patients were negative for HIV-1, hepatitis B and C, and syphilis.

**Table:** Clinical signs and symptoms reported at enrollment visit in study of persistence of Zika virus in different body fluids of persons with confirmed infection, Brazil*

Patient ID	Age, y/sex	Symptoms at enrollment
191001	21/M	None (asymptomatic household contact)
191002	25/M	Erythema and vesicular rash, nasal congestion, sweating, upper limb muscle spasms
291001	22/F	Itchy macular rash, conjunctival hyperemia, prostration, chills, taste alteration, lower back pain, arthralgia, periarticular edema, nausea, irritability
151001	38/M	Itchy macular rash, headache, photophobia, retro-orbital pain, burning eyes, arthralgia (wrists, metacarpals, and phalanges), tingling hands
251001	31/F	Itchy macular rash, conjunctival hyperemia, anorexia
251002	36/F	Itchy macular rash, headache, conjunctival hyperemia, arthralgia (shoulders, elbows, wrists, knees, ankles), abdominal pain, periarticular edema (ankles)
251003	43/F	Itchy macular rash, photophobia, retro-orbital pain, oropharyngeal pain, arthralgia (elbows and ankles)
251005	19/F	Itchy macular rash, fever, headache, photophobia, conjunctival hyperemia, retro-orbital pain, muscle weakness, numbness, irritability, appetite loss, nausea
251006	65/F	Itchy macular rash, fever, headache, photophobia, arthralgia (wrists, phalanges, heel, cervical spine), muscle weakness, prostration, numbness, tingling, drowsiness, abdominal pain, nausea
251007	32/F	Itchy macular rash, fever, headache, retro-orbital pain, photophobia

*Reported signs and symptoms were registered after an initial rash episode following laboratory confirmation of Zika virus infection. All signs and symptoms were registered <2 d after the initial rash (except for 1 asymptomatic contact patient). Fever (>38°C) was any febrile episode reported within 30 d before the medical examination. ID, identification.

For all patients, Zika virus RNA was detected >1 time in different body fluids. The longest interval for Zika virus–positive results by rRT-PCR in plasma and urine was 14 days after symptom onset; median cycle threshold value (C_t_) was 34.92 in plasma (interquartile range [IQR] 33.57–36.78) and 31.57 in urine (IQR 30.34–34.92). The longest duration of persistence in saliva was 21 days. Zika virus RNA was detected in rectal swab samples from all patients (median C_t_ 34.36 [IQR 30.08–36.07]); positive results were obtained at >1 time for 4 patients. Vaginal swab sample results were positive for 2 patients (Figure). We attempted virus isolation in Vero E6 cells from all rectal swab specimens positive by rRT-PCR. After filtering samples through a 0.22-μm syringe filter, a rectal swab sample from patient 191002 (at 7 days after symptom onset) was positive in Vero E6 cells, inducing cytopathic effect and returning a C_t_ of 28.77 after 6 days of incubation at 37°C.

## Conclusions

Our detection of Zika virus RNA in human rectal swab samples demonstrates the presence of virus RNA in the anorectal mucosa of naturally infected patients. Because the anorectal mucosa is a major entry site for HIV-1 and other sexually transmitted disease organisms (*9*), this finding may have implications for Zika virus transmission. Direct contact with infected mucosa could present a risk for virus transmission. Moreover, recovery of infectious Zika virus from anorectal mucosa samples may be associated with active virus replication in this body compartment, implicating its permissiveness to Zika virus infection.

Recently, 2 studies explored the mucosa as an experimental entry site for Zika virus. Macaques exposed to high doses of the virus by direct inoculation on palatine tonsils, nasal mucosa, and conjunctival mucosa became infected, whereas virus-naive animals exposed to saliva from Zika virus–infected macaques, containing a 20-fold lower virus concentration, remained uninfected (*6*). In mice, intra-anal inoculation resulted in viremia, replication in multiple organs, and Zika virus RNA detection in feces (*5*). However, it seems that high viral loads are required for successful experimental infection through mucosal tissues. In humans, a report of a man infected through anal intercourse implies that the anorectal mucosa is an entry site for Zika virus (*10*). Also, a rapidly progressive fatal case, with secondary nonsexual transmission to a close contact who reported having had no contact with blood or other body fluids except tears from the original patient, raised the hypothesis of Zika virus transmission through mucosa (*11*). Although mucosal tissues have been extensively explored as a potential site of Zika virus infection, the amount of infectious particles in these tissues remains unclear.

In patients with yellow fever, mucocutaneous bleeding with virus presence has been reported (*12*). However, no mucosal bleeding was observed in the patients we report, despite extensive medical examination. In addition, in 5 of 9 patients, the virus had already cleared from the blood at the time of anorectal detection, which supports the concept of local virus replication. Our finding of Zika virus in the anorectal mucosa of naturally infected persons may influence the recommendations for prevention of Zika virus transmission. We suggest the use of rectal swabbing, a noninvasive method, for diagnosing infection with Zika virus, among other emerging viruses (*13*).
